# The Impact of the Closure and Reopening of Golf Courses in the United Kingdom on Wellbeing During the COVID-19 Pandemic: A Multi-Study Approach

**DOI:** 10.3389/fspor.2021.622171

**Published:** 2021-02-02

**Authors:** Graeme G. Sorbie, Alexander J. Beaumont, Ashley K. Williams, Jonathan Glen, Scott M. Hardie, David Lavallee

**Affiliations:** ^1^Division of Sport and Exercise Sciences, School of Applied Sciences, Abertay University, Dundee, United Kingdom; ^2^School of Science, Technology and Health, York St John University, York, United Kingdom

**Keywords:** coronavirus, exercise, physical activity, SARS-CoV-2, sport

## Abstract

The purpose of this multi-study was to assess what impact the closure and reopening of golf courses had on: personal competence; sense of belonging; enjoyment; self-esteem; self-confidence; resilience; social connections; wellbeing and life satisfaction (hereafter referred to collectively as “key variables of interest”) during the COVID-19 pandemic. Golfers (Study 1: *n* = 298, Study 2: *n* = 124) ≥16 years old residing in the UK participated in this study which collected data using online surveys. Study 1 was conducted during a period of quarantine restrictions (4–12th May 2020), whilst study 2 took place following the easing of the COVID-19 quarantine restrictions (6–14th July 2020). Within study 1 and study 2, key variables of interest levels were also collected to investigate the association with golf related activities. The findings of study 1 highlighted that negligible and non-significant correlations were observed between golf physical and sedentary activities and key variables of interest (*r* < 0.3, *p* > 0.05) except sense of belonging and sedentary golf activities (*r* = 0.178, *p* = 0.003). Study 2 highlighted that sense of belonging, enjoyment and wellbeing were significantly associated with outdoor golf course activity (*r* = 0.186–0.252, *p* ≤ 0.05). Furthermore, when comparing study 1 and study 2, sense of belonging and life satisfaction significantly improved (*p* < 0.05, *d* = 0.2). Based on these findings, playing golf on outdoor golf courses appears to be positively related to sense of belonging, enjoyment and wellbeing. Also, with the reopening of golf courses life satisfaction improved which, together, highlights the beneficial impact that outdoor golf can impart.

## Introduction

Golf is played in 206 countries worldwide (The Royal Ancient, 2019), including the United Kingdom (UK), with 870,996 registered golfers (Lange, 2019). Golf is normally played outdoors on 9 or 18-hole courses and requires individuals to perform intermittent bursts of walking and multiple golf shots of varying distances (Versteegh et al., 2008). As a result of these aspects, golf is a popular sport across varying age groups and enables individuals with varying levels of fitness and mobility to participate (Arkkari et al., 2000; Murray et al., 2017).

Golf provides individuals with opportunities to increase or maintain light to moderate intensity physical activity (PA) levels (Luscombe et al., 2017). Golf is predominantly recognised as a moderate intensity activity, with a general metabolic equivalents (METs) of 4.8 (Ainsworth et al., 2011). Accordingly, golf can provide possible health benefits for those individuals who participate (Murray et al., 2017; Sorbie et al., 2020b), whilst also facilitating the opportunity to improve mental health and wellness (Murray et al., 2017; Breitbarth and Huth, 2019). Specifically, golf offers the prospect to improve social relations, sense of belonging, self-esteem, life satisfaction, personal competence, and personal wellbeing (Stenner et al., 2016; Wheatley and Bickerton, 2017; Sorbie et al., 2020b). In addition to the above, golf is normally played outdoors, which can also promote life satisfaction due to the outdoor environment (Zhang et al., 2014; Silva et al., 2018).

On March 23rd 2020, golf courses in the UK closed for between 8 and 10 weeks (the variation was dependent on different UK countries) as a result of the COVID-19 pandemic caused by SARS-CoV-2 coronavirus (United Kingdom Government, 2020). Although golf courses were closed, it was possible for individuals to perform golf-related activities within their home environment. We recently reported on golf-related engagement pertaining to physical (e.g., practising full golf swings, chipping, and putting) and sedentary (e.g., watching TV and online tutorials and listening to podcasts) activities during an 8-day period of restricted movement as a result of the COVID-19 pandemic (Sorbie et al., 2020a). Forty-eight percent of golfers completed physical golf-related activities within the home environment during a period of quarantine (4–12th May 2020). The most commonly-performed sedentary golf activity was watching golf on television (71%). Whilst informative, the implications of these activities during a period of restricted movement are not known, particularly in relation to personal competence, sense of belonging, enjoyment, self-esteem, resilience, social connections, and wellbeing. Indeed, behavioural strategies to alleviate the impacts of psychosocial stresses during quarantine restrictions have recently been recommended (Ricci et al., 2020). Moreover, the reopening of golf courses within the UK on 13–29th May 2020 enabled the opportunity to investigate the association between markers of personal wellbeing and golf-related activity both at home and on outdoor golf courses, importantly within the same individuals.

Taken together, the closure and reopening of golf courses during a unique and unprecedented period presented an opportunity for insightful implications for golf to be investigated. Therefore, the overarching purpose of this multi-study was to assess what impact golf-related engagement during the COVID-19 quarantine restrictions had on: personal competence; sense of belonging; enjoyment; self-esteem; self-confidence; resilience; social connections; wellbeing; and life satisfaction (hereafter referred to collectively as “key variables of interest”). In order to achieve this, we conducted studies within two distinct and significant time periods during the COVID-19 pandemic.

Study 1 aimed to ascertain the correlations between golf-related activities performed within the home environment and the key variables of interest during the COVID-19 quarantine restrictions (4–12th May 2020). It was hypothesised that home-based golf activity (physical and sedentary) would be positively associated with all key variables of interest.

Study 2 aimed to first ascertain the correlations between golf-related activities performed on golf courses and driving ranges/practise areas and the key variables of interest following the easing of the COVID-19 quarantine restrictions (6–14th July 2020). It was hypothesised that home-based (physical and sedentary) golf activities and those on golf courses and at driving ranges/practise areas would be positively associated with all key variables of interest. Secondly, study 2 aimed to compare key variables of interest between studies 1 and 2 with hypothesised improvements.

## Methods

### Participants Inclusion Criteria

In order to be eligible for the present studies, participants were required: to be 16 years of age or older; to consider themselves either a social, handicap or professional golfer; to reside within the UK and have played at least two rounds of golf in 2019. A minimum of two rounds of golf in 2019 was added within the eligibility criteria to ensure golfers could be categorised as a social golfer. Full ethical approval was granted from Abertay University School of Health Sciences prior to data collection.

### General Methodological Procedures

The same methodological procedures were used for both studies 1 and 2. Online surveys were used to collect data between 4 and 12th May 2020 (Study 1) and then subsequently between 6 and 14th July 2020 (Study 2). The surveys contained questions relating to three strands. Firstly, participants answered questions relating to what golf activities they performed during these two time periods. Golf-related questions included: what physical golf activities (i.e., full golf shots, chipping, putting, physical virtual reality golf games, participating in golf coaching sessions) were performed and what sedentary golf activities (i.e., reading golf magazines, listening to golf related podcasts or audiobooks, watching golf on TV) were engaged with. The days per week and time spent participating in these activities in the previous 7 days were recorded. Only activity bouts of at least 10 min were recorded. Specific to study 2, in accordance with re-opening of golf-courses, questions were asked relating to golf activity on courses, driving ranges, and practise areas between 6 and 14th July 2020. Data were also collected pertaining to the days per week and time spent participating in these activities in the previous 7 days.

Secondly, PA was measured using the International Physical Activity Questionnaire short form (IPAQ-SF). Although the recommended age range for using the IPAQ-SF is 15–69 years of age, we elected to use this assessment tool for older adults given the anticipated range in ages relating to golfers. The IPAQ-SF has previously been shown to have acceptable reproducibility (Craig et al., 2003), including older adults (Tran et al., 2013). We intended to use PA data derived from IPAQ-SF as a potential covariate to golf-related activity, depending on associated changes in PA from study 1 to study 2. In addition to the standard example, we supplied additional exemplar activities with a focus on typical behaviours likely to be experienced during restricted movement conditions to guide participants in accordance with known metabolic equivalents (METs) for each category of intensity (Ainsworth et al., 2011). Similarly, we calculated golf-related PA on outdoor courses and at driving ranges/practise areas using a 7-day recall in line with the IPAQ-SF.

Thirdly, participants completed a total of 18 questions relating to: personal competence (McAuley and Duncan, 1989); sense of belonging (Postmes et al., 2013); self-esteem (Robins et al., 2001); self-confidence (Bandura, 2006); resilience (Ungar and Liebenberg, 2011); social connections (Perlman and Peplau, 1981); wellbeing (Abdel-Khalek, 2006) and life satisfaction (Office of National Statistics). These questions have been commonly used within the respected areas, are validated measures and were answered on a 5-point scale, which ranged from strongly disagree (1) to strongly agree (5). Internal consistency was determined for variables of interest which were derived from multi-item questions, including personal competence (Cronbach's alpha = 0.80), resilience (Cronbach's alpha = 0.83), and social connections (Cronbach's alpha = 0.81).

### Data Analysis and Qualification of Activity

Physical activity data were processed in accordance with IPAQ-SF recommendations for each of the three intensities (vigorous, moderate, and walking) in order to calculate the MET.min^−1^.week^−1^. Total activity was then calculated to represent the sum of all intensities. For an extended and detailed method of data processing used in these studies, see Supplementary Material 1. The same processes were used to determine golf MET.min^−1^.week^−1^ on golf courses and at driving range/practise areas within the last 7 days. The METs of general golf on golf courses were deemed to be 4.8 and at the driving range/practise area, 3.0.

### Statistical Analysis

Statistical analyses were performed using Jamovi (Version: 1.2.12) (The Jamovi Project, 2019). All data were measured for normality using the Shapiro-Wilk test. For study 1 and 2, all data were not normally distributed; therefore, non-parametric Spearman's Rank Correlations were conducted to determine relationships between golf-related activity and key variables of interest. Correlation coefficients of 0–0.3 were categorised as negligible, 0.3–0.5 low, 0.5–0.7 moderate, 0.7–0.9 high, and 0.9–1 very high (Hinkle et al., 2009). Based on data collected in study 1, Cronbach's alpha was used to assess internal consistency for personal competence, resilience, and social connections which had multi-item questions.

For study 2, all data were not normally distributed; therefore, non-parametric Spearman's Rank Correlations were conducted to determine relationships between golf-related activity conducted on golf courses or driving ranges/practise areas and key variables of interest. In addition, non-parametric Wilcoxon singed-rank tests were carried out to compare golf-related engagement within the home environment, PA, and key variables of interest between study 1 and study 2. All data are presented as mean ± standard deviation (SD), *p*-value and effect sizes using Cohen's d (Cohen, 1988). Effect sizes of <0.2 were considered negligible, 0.2–0.5 small, 0.5–0.8 medium, and >0.8 large (Cohen, 1988). In all instances, *p* ≤ 0.05 was considered to be statistically significant.

## Results and Discussion

The results for study 1 are presented below, followed by a relevant discussion for study 1. Study 2 is presented in the same format as study 1. Following the results and discussion for study 1 and study 2, a general discussion is presented at the end of this section.

### Study 1—Results

A total of 298 golfers (14% Females; 86% Males) volunteered to participate in study 1. Golfers ranged in age from 16 to 89 years (Mean ± SD: 53 ± 15 years). Ninety-five percent had a handicap index; 3% were social golfers and 2% were professional golfers. The handicap index ranged from 0 to 50 (Mean ± SD: 14 ± 8 handicap index). At the time of the restricted movement period (4–12th May 2020), 34% were not working, 30% were working from home, 18% were working as normal, and 18% were retired. Of the included golfers for study 1, individuals had completed 73 ± 26 (range 2–250) rounds of golf in 2019. All golfers provided informed written consent before participating in study 1 and study 2.

Table 1 provides Spearman's correlation coefficients and *p*-value for correlations between golf-related engagement questions and key variables of interest. Negligible and insignificant correlations were observed between physical golf activities and all key variables of interest (*r* = −0.084–0.088, *p* > 0.05). Sedentary activities within the home environment were significantly associated with sense of belonging (*r* = 0.178, *p* = 0.003). Negligible and insignificant correlations were observed between all other key variables of interest and sedentary golf activities (*r* = −0.115–0.079, *p* > 0.05). For extended descriptive data relating to the key variables of interest and physical and sedentary activities, see Supplementary Material 2.

**Table 1:** Spearman's correlation coefficients (*r*) and *p*-value for golf-related engagement questions when measured against key variables of interest in study 1 (during the covid-19 quarantine restrictions).

**Golf-related questions**	**Correlation analysis**	**Key variables of interest**								
		**Personal competence**	**Sense of belonging**	**Enjoyment**	**Self-esteem**	**Self-confidence**	**Resilience**	**Social connections**	**Wellbeing**	**Life satisfaction**
Physical golf activities	Spearman's *r*	0.047	0.088	0.071	−0.058	−0.084	−0.054	−0.007	0.004	−0.025
	*p*-value	0.436	0.114	0.242	0.334	0.161	0.370	0.908	0.943	0.682
Sedentary golf activities	Spearman's *r*	0.069	0.178	0.067	0.011	0.079	−0.001	−0.013	−0.019	−0.093
	*p*-value	0.244	**0.003**	0.264	0.848	0.185	0.992	0.833	0.754	0.123

*Bold values indicate statistical significance. Significance granted at p < 0.05*.

### Study 1—Discussion

As a result of the majority of the correlations between golf-related activity and key variables of interest being negligible and non-significant, the hypothesis for study 1 was rejected. This contrasts with existing work, where it has been previously reported that golf offers the prospect to improve social relations, sense of belonging, self-esteem, life satisfaction, and personal competence which, in turn, can lead to an overall enhancement in personal wellbeing (Stenner et al., 2016; Wheatley and Bickerton, 2017; Sorbie et al., 2020b). Initially it was assumed that our disparate findings to others could be a result of the strict quarantine restrictions that were in place during the data collection period of study 1 (United Kingdom Government, 2020). However, we recently reported that the common physical golf activities performed within the home environment included full golf swings, chipping, and putting (Sorbie et al., 2020a). Although these physical golf activities form a part of the game of golf, these skills do not replicate the outdoor environment that golf courses offer, as they do not include the walking element of the sport and the social interactions the sport provides. These aspects have been shown to promote personal wellness (Fox, 1999; Silva et al., 2018); therefore performing these physical golf activities within the home environment could aid skill development, but is unlikely to impact key variables of interest investigated within this study.

Moreover, we observed that sedentary golf activity and sense of belonging were significantly and positively associated. This suggests that those who engaged in sedentary golf activities for longer periods of time presented with a greater sense of belonging, even during a time when significant quarantine restrictions were imposed. We recently reported that the most commonly-performed sedentary golf activities during the COVID-19 quarantine restrictions were watching golf on television and watching online tutorials (Sorbie et al., 2020a). In support, it has been shown in other sports that spectating through media such as television can increase or create a sense of belonging for individuals (Williams, 2007). Whilst engagement in sedentary activities may provide a sense of belonging, these activities are likely conducted without face-to-face interaction, which was reduced given the concurrent movement restrictions. Accordingly, this may provide some insight to the lack of association observed between sedentary golf engagement and social connexion. These results suggest that engaging with golf on television or through online tutorials can provide individuals with a sense of belonging during a period of strict quarantine restrictions and with limited social interactions.

### Study 2—Results

A total of 124 golfers (17% Females; 83% Males) volunteered to participate in study 2. These golfers were recruited from the same pool of golfers that participated in study 1. Golfers ranged in age from 20 to 89 years (Mean ± SD: 54 ± 15 years). Ninety-four percent had a handicap index; 5% were social golfers and 1% were professional golfers. The handicap index ranged from 0 to 50 (Mean ± SD: 14 ± 9 handicap index). Of the included golfers for study 2, individuals had completed 82 ± 47 (range 2–250) rounds of golf in 2019.

Table 2 provides Spearman's correlation coefficients and *p*-value for correlations between golf-related engagement questions and key variables of interest obtained from study 2. In relation to golf activity on golf courses (MET.min^−1^.week^−1^), sense of belonging (*r* = 0.186, *p* = 0.041), enjoyment (*r* = 0.234, *p* = 0.010), and wellbeing (*r* = 0.252, *p* = 0.005) were significantly associated with these types of golf activity. All other key variables of interest and golf activity on golf courses (MET.min^−1^.week^−1^) and golf practice (MET.min^−1^.week^−1^) performed at the driving range/practice area were negligible and not significantly related (*r* = −0.084–0.171, *p* > 0.05).

**Table 2:** Spearman's correlation coefficients (*r*) and *p*-value for golf-related engagement questions obtained from study 2 (following the easing of COVID-19 quarantine restrictions) when measured against key variables of interest.

**Golf-related questions**	**Correlation analysis**	**Key variables of interest**								
		**Personal competence**	**Sense of belonging**	**Enjoyment**	**Self-esteem**	**Self-confidence**	**Resilience**	**Social connections**	**Wellbeing**	**Life satisfaction**
Golf activity (MET.min^−1^.week^−1^)	Spearman's *r*	0.091	**0.186**	**0.234**	0.093	0.026	0.148	0.138	**0.252**	0.171
	*p*-value	0.314	**0.041**	**0.010**	0.305	0.775	0.102	0.128	**0.005**	0.059
Golf practise (MET.min^−1^.week^−1^)	Spearman's *r*	−0.069	−0.042	−0.028	−0.084	−0.034	0.091	−0.021	−0.083	−0.046
	*p*-value	0.470	0.667	0.773	0.376	0.726	0.340	0.826	0.384	0.629
Physical Golf Activities	Spearman's *r*	0.056	**0.226**	−0.013	0.053	0.049	0.083	0.041	−0.001	−0.013
	*p*-value	0.541	**0.014**	0.885	0.566	0.592	0.365	0.655	0.995	0.855
Sedentary Golf Activities	Spearman's *r*	0.076	**0.277**	0.027	0.092	0.048	0.116	0.027	−0.032	0.073
	*p*-value	0.405	**0.002**	0.767	0.312	0.599	0.203	0.772	0.729	0.424

*Bold values indicate statistical significance. Significance granted at p < 0.05*.

Physical and sedentary golf activities within the home environment were significantly associated with sense of belonging (*r* = 0.226, *p* = 0.014, *r* = 0.277, *p* = 0.002). Negligible and insignificant correlations were observed between physical and sedentary golf activities within the home environment and all key variables of interest (*r* = −0.032–0.116, *p* > 0.05) (Table 2).

When comparing key variables of interest between studies 1 and 2, sense of belonging (*p* = 0.044, *d* = 0.167) and life satisfaction (*p* = 0.026, *d* = 0.223) significantly increased. No statistical significance was reported for all other key variables of interest between studies 1 and 2 (*p* > 0.05) (Table 3).

**Table 3:** Key variables of interest between study 1 and study 2 using a 5-point Likert scale with *p*-value and Cohen's d effect sizes.

**Key variables of interest**	**Study 1**	**Study 2**	***p*-value**	**Effect size (Cohen's d)**
Personal competence (*n* = 124)	2.77 ± 0.83	2.85 ± 0.77	0.162^a^	0.1
Sense of belonging (*n* = 120)	3.50 ± 1.08	3.67 ± 1.01	**0.044**^a^	**0.2**
Enjoyment (*n* = 120)	4.42 ± 1.07	4.34 ± 0.99	0.332^a^	0.1
Self-esteem (*n* = 124)	3.55 ± 1.14	3.55 ± 1.03	0.938^a^	0.0
Self-confidence (*n* = 123)	3.83 ± 1.07	3.90 ± 1.00	0.387^a^	0.1
Resilience (*n* = 124)	3.66 ± 0.85	3.59 ± 0.74	0.093^a^	0.1
Social connexion (*n* = 124)	3.65 ± 0.91	3.80 ± 0.92	0.057^a^	0.2
Wellbeing (*n* = 122)	3.68 ± 0.99	3.78 ± 0.94	0.508^a^	0.1
Life satisfaction (*n* = 121)	3.52 ± 1.11	3.79 ± 0.91	**0.026**^a^	**0.2**

*Data are mean ± SD. Bold values indicate statistical significance*.

a *Non-normally distributed analysis. Significance granted at p < 0.05*.

Physical golf activities within the home environment significantly reduced when comparing studies 1 and 2 (*p* < 0.001, *d* = 0.425), whereas no significant difference was observed between sedentary golf activities between studies 1 and 2 (*p* = 0.550, *d* = 0.126) (Table 4). Furthermore, moderate and vigorous PA significantly reduced between studies 1 and 2 (*p* < 0.001, *d* = 0.181, *p* = 0.024, *d* = 0.203), whereas no significant difference was reported for light PA between studies 1 and 2 (*p* = 0.342, *d* = 0.048). For extended descriptive and statistical data relating to PA, see Supplementary Material 2.

**Table 4:** Golf-related activities between study 1 and study 2.

**Golf-related activity**	**Study 1**	**Study 2**	***p*-value**	**Effect size (Cohen's d)**
Physical (min.week^−1^) (*n* = 113)	88 ± 166	27 ± 54	**<0.001**^a^	**0.4**
Sedentary (min.week^−1^) (*n* = 117)	151 ± 217	200 ± 367	0.550^a^	0.1

*Data are mean ± SD. Bold values indicate statistical significance*.

a *Non-normally distribute analysis. Significance granted at p < 0.05*.

### Study 2—Discussion

Due to multiple correlations observed between golf-related activity and key variables of interest, the original hypotheses for study 2 were partially accepted. Specifically, study 2 reported significant and positive correlations between golf course activity (MET.min^−1^.week^−1^) and enjoyment, wellbeing and sense of belonging. This demonstrates the advantages of spending more time on golf courses playing golf. These positive findings are in agreement with existing research that has investigated the impact that golf has on various psychosocial markers of health (Murray et al., 2017; Breitbarth and Huth, 2019). Collectively, these findings reinforce the importance of engaging with golf activities on outdoor golf courses. Further support for actually playing golf on outdoor courses as the mediator is clear when considering that practice/driving range activity (MET.min^−1^.week^−1^) was not associated with any markers of belonging, enjoyment, competence, resilience, social connections, wellbeing, or life satisfaction. Therefore, although practice area/driving range activities provide opportunities to enhance skill level and increase PA levels, these types of activities were not related to key variables of interest herein.

Importantly, there were also insignificant correlations between golf course activity (MET.min^−1^.week^−1^) and other key variables of interest, including social connections, personal competence, and self-confidence. It has been previously highlighted that golf is associated with increased social connections (Berlin and Klenosky, 2014); however, the disagreement in this study may be a result of the partial restrictions imposed on the return of golf during the time of study 2. For example, social distancing, playing with a limited number of golfers and no hand shaking at the end of a round (England Golf, 2020). This may provide a plausible explanation as to why social connections and golf course activity were not significantly associated.

In relation to personal competence and self-confidence, these measures were not significantly associated with golf course activity (MET.min^−1^.week^−1^). These results could be due to the extended period that golf courses were closed, which resulted in golfers being unable to play on outdoor courses. Specifically, during the initial restrictions set by the UK government, golf courses were closed for 8–10 weeks (United Kingdom Government, 2020). It is possible that this absence from golf courses may have imposed a negative effect on personal competence and self-confidence upon returning to the sport. Although under different conditions, this is supported by previous literature that highlights athletes' competence and confidence is adversely impacted when injured for a prolonged period of time (Clement et al., 2015).

## General Discussion

The aim of this multi-study was to investigate the impact of golf-related engagement on: personal competence; sense of belonging; enjoyment; self-esteem; self-confidence; resilience; social connections; wellbeing and life satisfaction during the COVID-19 pandemic within a cohort of golfers. The principle findings were that: (1) during quarantine restrictions (study 1) there were negligible correlations between golf activity within the home environment and key variables of interest.; (2) Following the reopening of golf courses (study 2), positive correlations were observed between golf course activity (MET.min^−1^.week^−1^) and sense of belonging, enjoyment and wellbeing; (3) When considering both studies and the transition from quarantine restrictions to being able to play outdoor golf, significant improvements were observed in sense of belonging and life satisfaction. Taken collectively, this multi-study provides insight into golf-related activities during an unprecedented time during a global pandemic and how these can facilitate superior perceptions of sense of belonging, wellbeing and life satisfaction when golf is conducted on outdoor courses.

When considering both studies and the transition from having quarantine restrictions in place to being able to play golf on golf courses, small yet significant improvements were observed in relation to sense of belonging. Although many golfers during studies 1 and 2 were able to engage in physical golf activities within their home environment, these skills do not fully-reflect the sport of golf. This is supported by the lack of correlations between physical and sedentary golf activities within the home environment and key variables of interest in both studies 1 and 2. In particular, the skills that were being performed within the home environment do not reflect the outdoor environment that golf courses offers, including the element of walking and the competitive nature of the sport; accordingly, it is likely attributed to the act of play on outdoor golf courses, which agrees with previous research. Specifically, Stenner et al. (2016) reported that golf, within the natural golf environment, can have a positive impact on an individual's sense of belonging; therefore, the reopening of golf courses most likely explains the significant increase in sense of belonging between studies 1 and 2.

Additionally, life satisfaction also increased between studies 1 and 2. The reopening of golf courses alongside less-restrictive quarantine measures may have facilitated this improvement. Playing golf on courses enables individuals to play the sport in a natural environment, which is known to increase life satisfaction (Zhang et al., 2014). Whilst we cannot completely disentangle PA from physical golf activity, we are confident that the improved life satisfaction reflects the re-opening of golf courses and less-restrictive measures, as opposed to changes in PA levels. Indeed, total PA (MET.min^−1^.week^−1^) levels (which did not include golf activity) significantly reduced, therefore, the previously reported psychological benefits of increased PA (Hartfiel et al., 2011) would not appear to be a principle factor in the enhancement of life satisfaction; although the contrary is also true, and it must be recognised that concomitant alterations in social interaction could have contributed. Nonetheless, this observation may indicate a situational change in life satisfaction, with concurrent increases in outdoor golf course activity being performed. The positive association between golf course activity (MET.min^−1^.week^−1^) and wellbeing, as reported in study 2, however, may better reflect that specific time period of being able to play golf. Therefore, when taken together we recommend that, where possible, golf should be played on outdoor courses even if there are future quarantine restrictions put in place by governments to ensure improved life satisfaction, and individuals should be encouraged to spend more time on golf courses (MET.min^−1^.week^−1^) for an association with greater wellbeing. These findings may also be comparable to other sports that display similarities to golf in regards to the required METs score between 4.0 and 5.0 for activities that include but are not limited to: archery, basketball shooting, cricket, table tennis, track and field throwing events, and doubles tennis (Ainsworth et al., 2011).

When comparing both studies, no significant differences were observed in all other variables of interest. However, social connections tended to increase from study 1 to study 2 (Table 3). Although we would expect that the reintroduction of golf courses would significantly enhance social connections based upon previous research (Berlin and Klenosky, 2014), strict restriction measures were still in place when golf courses reopened. These restrictions included restricted locker room and clubhouse access (England Golf, 2020). Although golfers were able to play on golf courses with other golfers, these strict restrictions may help to explain why the social connections between studies 1 and 2 were not statistically significant, yet a small effect was observed. It would be of interest to see if social connections are enhanced if and when all restrictions are removed. The present findings may be applicable to other sports that are dependent on the closure and reopening of sporting facilities.

No significant differences were found in personal competence, enjoyment, self-esteem, and self-confidence when comparing studies 1 and 2. We feel that these findings could be a result of golfers being unable to play on golf courses for an extended period of time. As a result, this time away from the sport may have impacted on performance levels, which may have resulted in no change being observed in personal competence, enjoyment, self-esteem, and self-confidence. Indeed, when comparing the present findings with previous research, time away from sporting competition in sports such as football and baseball has been previously shown to have an impact on these measures (Clement et al., 2015). Future research may be required in order to measure the impact that personal competence, enjoyment, self-esteem, and self-confidence can have when golfers have been playing for an extended period without an unanticipated time away from the sport.

Strengths of this multi-study include the timeframe that the surveys were implemented. This ensured that the UK government guidelines in relation to golf were captured at similar levels for all individuals across the two studies. In addition, the golfers within this multi-study are representative of the numbers of registered golfers in the UK, including age (Sorbie et al., 2020b), golf handicap index (Golf Care, 2016), and gender (Lange, 2019). In relation to gender, 81% of registered golfers in the UK are male and 12% are female (Lange, 2019). This distribution in gender is representative of golfers that participated within this multi-study (Study 1: 86% Male and 14% Female, Study 2: 83% Male and 17% Female). Additionally, an important and novel aspect of this multi-study is the follow-up nature and collection of data within the same individuals during an unprecedented time.

As a result of this multi-study being conducted during the COVID-19 pandemic, the findings should be contextualised as a result of the methodological limitations. Specifically, there were significant relaxations in quarantine restrictions during the data collection of study 1 and study 2, such as increased contact with family and friends (United Kingdom Government, 2020); therefore, it remains unclear to what degree golf participation contributes to the improvements in the measures within this study. In addition, the significant and positive correlations observed within studies 1 and 2 were categorised as negligible or small; however, we do anticipate that other uncontrollable factors associated with the pandemic may have influenced these relationships.

At the time of writing, it is uncertain if or when the COVID-19 pandemic will recede, and there may be a need for quarantine measures to be reintroduced at some stage. If this were to happen, there would likely be an impact on many sports. Based on the current findings, however, we would recommend that on-course golf activity should be introduced at an early stage of any restrictive period, with safety measures already having been put in place by governing bodies responsible for golf. In addition, the current study focused on the psychosocial benefits of participating in golf, which is a low to moderate PA, during the COVID-19 pandemic. Whilst we did not investigate age related differences, future research may wish to do so based on the different exercise intensities of walking an 18-hole golf course is experienced by young, middle-aged and elderly golfers (Broman et al., 2004). In addition, future research is required to investigate if the benefits of participating in this type of activity are translatable to other sports with similar intensities, as well as to investigate whether or not higher intensity sports further enhance psychosocial measures during an unprecedented period.

## Conclusion

The principle findings of this multi-study were that there were negligible correlations between golf activity within the home environment and key variables of interest. Following the reopening of golf courses, positive correlations were observed between golf course activity (MET.min^−1^.week^−1^) and sense of belonging, enjoyment and wellbeing. When considering both studies, significant improvements were observed in sense of belonging and life satisfaction, which may be crucial during the current pandemic, or even future pandemics. Accordingly, this study has provided insight during a global pandemic with regards to the association between golf activity conducted indoors and on outdoor courses, and the benefits of the latter on sense of belonging and life satisfaction.

## Data Availability Statement

The original contributions presented in the study are included in the article/Supplementary Material. Further inquiries can be directed to the corresponding author/s.

## Ethics Statement

The studies involving human participants were reviewed and approved by Abertay University. The patients/participants provided their written informed consent to participate in this study.

## Author Contributions

All authors listed have made a substantial, direct and intellectual contribution to the work, and approved it for publication.

## Conflict of Interest

The authors declare that the research was conducted in the absence of any commercial or financial relationships that could be construed as a potential conflict of interest.
