# Supplementary material for: The Impact of the Closure and Reopening of Golf Courses in the United Kingdom on Wellbeing During the COVID-19 Pandemic: A Multi-Study Approach
Source: Front Sports Act Living. 2021 Feb 2;3:622171. doi: 10.3389/fspor.2021.622171 (PMC7884885; doi:10.3389/fspor.2021.622171)
Supplement: Supplementary file 1 [file Table_1.pdf]

SUPPLEMENTARY MATERIAL 1

Processing of physical activity data:

For golf-related questions, only activity bouts of at least 10 minutes were considered, therefore, values of < 10 minutes per day were re-coded as ‘zero’, in addition to their associated days. In order to calculate total golf-related activities per week, number of days was multiplied by number of minutes for each category (physical golf activities, physical golf training and sedentary golf activities). In order to calculate total physical activity per week, number of days was multiplied by number of minutes exercise within the respective category per day. Following this, a MET-minute score for each intensity category was calculated by multiplying total physical activity per week by number of METs for that intensity category. The MET intensities used to score IPAQ categories were: vigorous (8 METs), moderate (4 METs) and walking (3.3 METs) (Oyeyemi et al., 2014). Data cleaning and processing were conducted in accordance with IPAQ recommendations (Guidelines for Data Processing and Analysis of the International Physical Activity Questionnaire (IPAQ) – Short and Long Forms, 2005). Time spent exercising < 10 minutes were re-coded to ‘zero’ minutes, in addition to their associated days irrespective of whether the days was left blank, ‘zero’ or ≥1. In the instance that ‘zero’ days was imputed and minutes left blank, the authors added a ‘zero’ for the minutes as no activity has already been stated. Cases were removed from analysis if, both days and minutes were left blank, ‘zero’ days was reported but with >10 minutes activity, ≥1 days was inputted but minutes were left blank or blank number of days but >10 minutes. Outliers were removed in the instance that the sum of vigorous, moderate and walking exercise per week exceeded 960 minutes. Finally, in the interest of normalising data distribution, values exceeding 3 hours or 180 minutes per day were truncated to 180 minutes. This approached facilitated a maximum of 21 hours of active per category within a week (i.e., 3 hours x 7 days).

Guidelines for Data Processing and Analysis of the International Physical Activity Questionnaire (IPAQ) – Short and Long Forms (2005).

Oyeyemi, A. L., Umar, M., Oguche, F., Aliyu, S. U., and Oyeyemi, A. Y. (2014). Accelerometer-determined physical activity and its comparison with the international physical activity questionnaire in a sample of Nigerian adults. *PLoS One* 9. doi:10.1371/journal.pone.0087233.
