# Supplementary material for: The Impact of the Closure and Reopening of Golf Courses in the United Kingdom on Wellbeing During the COVID-19 Pandemic: A Multi-Study Approach
Source: Front Sports Act Living. 2021 Feb 2;3:622171. doi: 10.3389/fspor.2021.622171 (PMC7884885; doi:10.3389/fspor.2021.622171)
Supplement: Supplementary file 2 [file Table_2.pdf]

SUPPLEMENTARY MATERIAL 2

Table A

Study 1 descriptive data using a 5-point Likert scale for key variables of interest.

**Key Variables of Interest**	**Study 1**
Personal competence (n= 297)	2.71 ± 0.84
Sense of belonging (n= 291)	3.48 ± 1.05
Enjoyment (n= 290)	4.48 ± 0.96
Self-esteem (n= 294)	3.53 ± 1.09
Self-Confidence (n= 294)	3.82 ± 1.00
Resilience (n= 297)	3.41 ± 0.53
Social connection (n= 296)	3.61 ± 0.90
Wellbeing (n= 293)	3.67 ± 0.98
Life Satisfaction (n= 290)	3.53 ± 1.05

Data are mean ± SD.

Table B

Study 1 descriptive data for physical activity levels.

**Physical Activity**	**Study 1**
Total $MET.min^{-1}.week^{-1}$ (n= 291)	2710.71 ± 2194.71
Light $MET.min^{-1}.week^{-1}$ (n= 287)	680.82 ± 841.47
Moderate $MET.min^{-1}.week^{-1}$ (n= 289)	1019.17 ± 972.72
Vigorous $MET.min^{-1}.week^{-1}$ (n= 281)	1063.63 ± 1549.47

Data are mean ± SD. MET = metabolic equivalent.

Table C

Study 1 descriptive data for golf-related activities.

**Golf-related Activity**	**Study 1**
Physical (min.week^{-1}) (n= 283)	86 ± 165
Sedentary (min.week^{-1}) (n= 285)	141 ± 229

Data are mean ± SD.

Table D

Physical activity between study 1 and study 2.

**Physical Activity**	**Study 1**	**Study 2**	**p-value**	**Effect size (Cohen's d)**
Total MET.min^{-1}.week^{-1} (n= 123)	2785.98 ± 2327.94	2087.70 ± 1917.42	**<0.001**[a]	**0.3**
Light MET.min^{-1}.week^{-1} (n= 123)	660.83 ± 869.37	614.66 ± 841.22	0.342[a]	0.0
Moderate MET.min^{-1}.week^{-1} (n= 123)	1332.85 ± 3066.01	759.51 ± 864.81	**<0.001**[a]	**0.2**
Vigorous MET.min^{-1}.week^{-1} (n= 115)	1111.65 ± 1796.41	758.26 ± 1265.14	**0.024**[a]	**0.2**

Data are mean ± SD. **Bold** values indicate statistical significance. [a] Non-normally distributed analysis. Significance granted at $p<0.05$.
